# Isosorbide as a Molecular Glass: New Insights into the Physicochemical Behavior of a Biobased Diol

**DOI:** 10.3390/molecules30224364

**Published:** 2025-11-11

**Authors:** Nadia Hammami, Stéphane Patry, Armand Soldera, Bruno Ameduri, Jean-Pierre Habas

**Affiliations:** 1 ICGM, Univ Montpellier, CNRS, ENSCM, 34000 Montpellier, France; nadia.hammami@umontpellier.fr (N.H.); stephane.patry@umontpellier.fr (S.P.); bruno.ameduri@enscm.fr (B.A.); 2 Department of Chemistry, University Sherbrooke, Sherbrooke, PQ J1K 2R1, Canada; armand.soldera@usherbrooke.ca

**Keywords:** isosorbide, hydrogen bond, calorimetry, thermostability, viscoelastic properties, molecular glass, cooperative molecular motions

## Abstract

This paper presents a study of the thermal and rheological properties of isosorbide, showing that its degradation temperature (around 100 °C) is much lower than values previously proposed in the literature. Furthermore, remarkable calorimetric and viscoelastic behaviors, with features usually observed in semi-crystalline systems are presented. The onset of the melting is measured at 45 °C, while a glass transition occurs at −45 °C, followed by cold crystallization. Wide-angle X-ray diffraction confirmed the coexistence of crystalline domains and an amorphous fraction, which behaves as a molecular glass, with an estimated crystallinity of approximately 70%. Thermogravimetric analyses conducted under both air and nitrogen and at multiple heating rates, in line with ICTAC recommendations, established the robustness of the 100 °C degradation onset. These findings provide new structure–property relationships for isosorbide and open up new avenues for further research and development in this area.

## 1. Introduction

**Figure 1:**
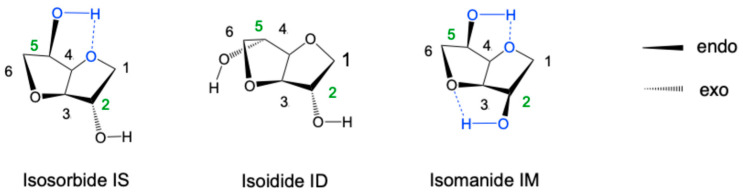
Chemical structures of the three isomeric isohexides (the blue color was used to highlight the chemical groups involved in the hydrogen bond ----).

Isohexides, also known as dianhydrohexitols, are a family of three chemical compounds characterized by a bicyclic skeleton carrying two hydroxyl (OH) groups and with the same raw chemical formula, C_6_H_10_O_4_. The linkage of two tetrahydrofuran-type forms the structure of these three diol rings with an opening angle of 120°, forming a concave shape like an open book or the letter “V.” The three isohexides differ in the relative orientation of their hydroxyl groups within the bicyclic structure, leading to three possible configurations [1,2] (Figure 1).

- Exo–exo or isoidide ID (or 1,4:3,6-dianhydro-L-iditol). In this arrangement, the hydroxyl hydrogens cannot form intramolecular hydrogen bonds with adjacent oxygens.
- Endo–endo or isomannide IM (or 1,4:3,6-dianhydro-D-mannitol). This symmetrical endo–endo conformation allows the formation of two intramolecular hydrogen bonds between the OH groups and the ether oxygens on each ring.
- Endo–exo or isosorbide IS (1,4:3,6-dianhydro-D-glucitol). One OH group is endo, allowing for a hydrogen bond as in IM, while the other is exo, making it more accessible to external reactive groups.

The endo or exo orientation of OH groups strongly influences their reactivity, and therefore that of the entire molecule (IS, ID, or IM). Studies have shown that the exo position is generally more accessible and reacts more readily with neighboring chemical units [3,4,5,6,7]. Consequently, ID (exo–exo) is usually the most reactive, IM (endo–endo) the least, and IS (endo–exo) intermediate. This trend is particularly observed under experimental conditions involving bulky substituents, where the lower steric hindrance of the exo OH favors reaction.

For IS, the coexistence of endo and exo OH groups allows regioselective reactions such as exo-alkylation, endo-alkylation, or tailored functionalization. However, this difference in reactivity can be a disadvantage in polymerization, where uniform reactivity of functional groups is often preferred.

**Figure 2:**
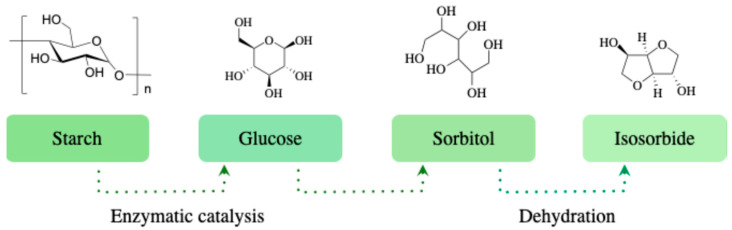
Production of isosorbide from starch in 3 steps.

Although the three isohexides described above share a similar bicyclic chemical framework defined by the orientation of their two hydroxyl groups, their origins and production methods differ significantly. Isosorbide IS, for example, is obtained from D-sorbitol, which is abundant in nature or can be derived from starch by enzymatic means (Figure 2). When isosorbide was discovered in the 1940s [8,9], it was produced in small quantities for pharmaceutical applications. The synthesis of isosorbide was then carried out on a larger scale, to meet mainly the needs of the cosmetics industry. From 2007 onwards, its industrial production reached a capacity of several thousand tons per year, enabling it to satisfy new markets and fields of application, such as organic chemistry, fine chemistry, and material chemistry [10]. In Europe, mass production of this diol is mainly carried out by Roquette Frères Company, the world’s leading sorbitol producer since 1954, which has mastered the double dehydration of this molecule to transform it into isosorbide. Separation and purification methods patented by Roquette enabled the marketing of POLYSORB^®^, a high-purity isosorbide specifically adapted for polymer manufacturing [11].

It is worth noting that new methods of producing isosorbide have been explored to avoid using food sources. For example, in 2017, Keskiväli et al. developed a new two-step method for synthesizing IS from cellulose, yielding 50% of the target [12]. The synthesis route for IM is quite different from that for IS. IM is produced from D-mannitol, which is derived from fructose, and is more expensive than glucose. On the other hand, ID is derived from L-iditol, a very rare compound in nature. This scarcity contributes to its high price and limits its potential for industrial use despite its superior performance compared to IS [4]. Recognizing these economic and ecological challenges, several research teams conducted studies to convert IM or IS to ID [1,4,13]. However, the reaction yields often remain low, and the processes are long and complex.

**Figure 3:**
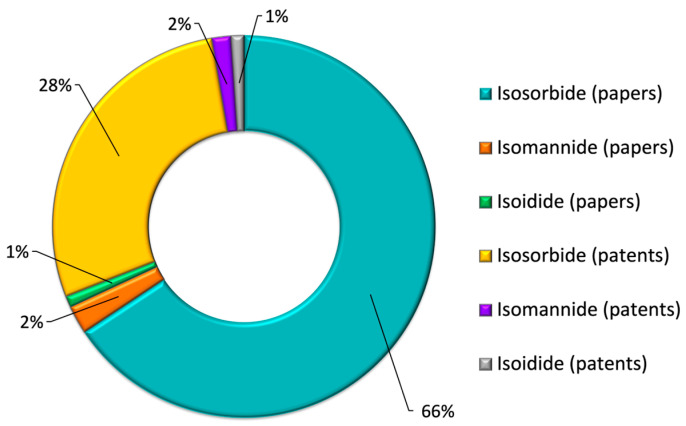
Scientific production statistics on IS, IM, and ID (‘Web of Science’, July 2025).

IS seems to be the most promising isohexide for industrial use due to its favorable combination of characteristics. It is abundant in raw material and offers superior performance compared to IM, all at a price significantly lower than that of II (at a ratio of 1:100). A review of the literature supports this observation, as isosorbide has been the most studied monomer by the scientific community. As of September 2024, 6222 scientific articles were devoted to IS, compared with 226 and 90 publications for IM and ID, respectively, over the same period (Figure 3). Isosorbide has been utilized as a building block for creating a wide range of molecules and materials through processes such as conventional alkylation, etherification, and esterification. These derivatives have been utilized in various applications across different sectors as described after [4,10,14,15].

### 1.1. Isosorbide-Based Molecules for Therapeutic Applications

**Figure 4:**
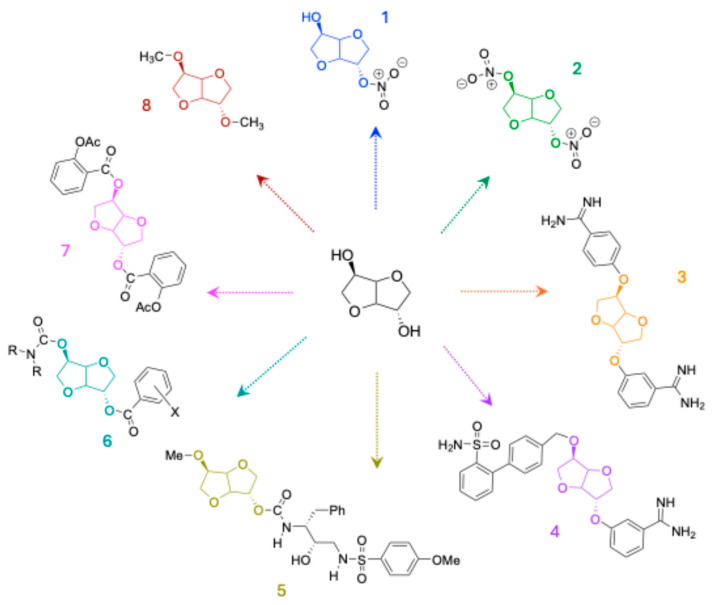
Representation of some molecules derived from isosorbide for medical or cosmetic applications.

The first IS-derived molecules were synthesized for medical and/or pharmaceutical applications (Figure 4). Mono-nitrate isosorbide (MNIS, compound 1 in Figure 4) and di-nitrate (DNIS, compound 2) were produced to be used as vasodilators to treat heart failure and angina pectoris [16,17]. Isosorbide-based bis- and mono-benzamidines (molecules 3 and 4) have also been developed as inhibitors of the serine protease enzyme [18].

Other molecules represented by compounds 5 and 6 are effective in limiting, if not controlling, the respective activities of human butyrylcholinesterase [19,20]. Isosorbide 2-aspirinate-5-salicylate (compound 7) is a prodrug of aspirin in human blood [21]. Finally, isosorbide dimethyl ether (DMI), compound 8, is a safe agricultural solvent that serves as a sustainable alternative to dipolar aprotic solvents, such as N-methyl-2-pyrrolidone (NMP) and N,N-dimethylformamide (DMF), due to its high solubilization capacity, making it suitable for both organic synthesis and the cosmetic industry [22].

### 1.2. Monomers Generated from Isosorbide Functionalization

**Figure 5:**
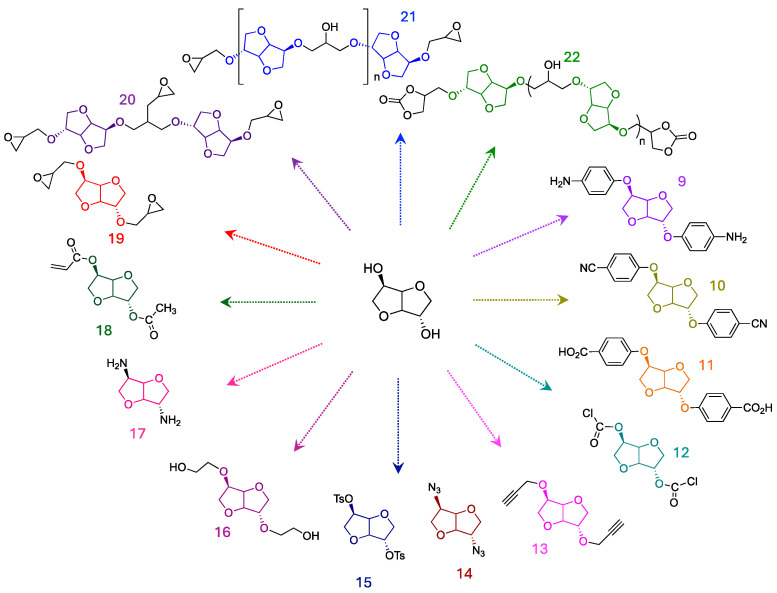
Representation of some monomers derived from isosorbide.

IS was also used to produce different monomers bearing specific chemical functions (tosylate, amine, azide, allyl, hydroxyl…) as precursors for a large variety of polymers, as shown in Figure 5.

Bortolussi et al. [23] have described a multi-step method for synthesizing IS-based aromatic derivatives. The technique involves replacing the IS hydroxyl groups with p-fluoronitrophenyl through nucleophilic substitution. The resulting dinitrophenyl isosorbide was then hydrogenated to produce diphenyl-amino-isosorbide (compound 9). Di(4-cyanophenyl) isosorbide (compound 10) is also obtained using a similar chemical pathway and then hydrolyzed to yield the corresponding diacid (compound 11) [24]. Such a compound is a possible substitute for terephthalic acid.

Bis-isosorbide chloroformate (BIC) compound 12 was synthesized by reacting IS with chlorophosgene [25]. This monomer can be used to produce various types of polymers, including polyesters, polycarbonates and polyamides.

Diazide and dialkyne isosorbide (compounds 13 and 14, respectively) were designed by Besset et al. for use as monomers involved in click chemistry step-growth polymerization. Isosorbide diazide required first to tosylate the alcohol groups of IS to give compound 15 followed by the nucleophilic substitution of the tosylate groups by sodium azide [26].

To enhance the low reactivity of isosorbide’s secondary hydroxyl groups compared with primary diols, bis(2-hydroxyethyl) isosorbide (compound 16) was synthesized by reacting isosorbide and ethylene carbonate with potassium carbonate. This primary diol derived from isosorbide is used as a raw material in the preparation of polyesters and polyurethanes [4].

Diamino isosorbide (DAIS) (compound 17) was prepared using the Gabriel synthesis, which involved three steps: tosylation, nucleophilic substitution with potassium phthalimide, and finally deprotection [27]. The yields for tosylation and deprotection were more than 80%, while the yield for the nucleophilic substitution of DTIS was only around 13%. The SN2 reaction on the exo-leaving group of isosorbide ditosylate was much less efficient than that on the endo-leaving group, which explains the low yield of the second step.

The versatile chemical functionalization of isosorbide has enabled the two-step synthesis of acetylated acrylic monomers such as (compound 18) by Reineek et al. [28]. Isosorbide dimethacrylate was synthesized using the method described by Sadler et al. [29], where methacryloyl chloride reacts with the two hydroxyl groups of isosorbide.

Numerous studies have explored the use of isosorbide as a substitute for harmful molecules in thermosetting resin formulations. One notable example is isosorbide diglycidyl ether (ISDGE) (compound 19), which was developed to replace the bisphenol A diglycidyl ether (DGEBA). Due to its aromatic core structure, DGEBA is recognized for its excellent mechanical and thermal properties and is utilized as a prepolymer in numerous epoxy formulations [30,31]. However, it is suspected of being an endocrine disruptor [32,33,34,35]. Replacing it with an isosorbide derivative featuring a central bicyclic skeleton is a promising area of research [36].

Pascault et al. tested two different methods to produce ISDGE [37]. The first method involves reacting isosorbide directly with epichlorohydrin under reduced pressure by adding a sodium hydroxide solution. This method produced linear and branched oligomers, designated as DGEDASn (compounds 20 and 21), in 98% yield. The second method involved two synthetic steps: the preparation of the allylic derivative of isosorbide, followed by its epoxidation with a peracid. This method needs additional purification steps to isolate the pure prepolymer, resulting in a lower yield (60%). However, it presents the advantage of producing ISDGE mainly.

Telechelic isosorbide dicyclocarbonates (compound 22) were synthesized by carbonating isosorbide diglycidyl ether oligomers (compounds 19, 20, and 21) [38]. The reaction of cyclocarbonates with amines offers a promising alternative for obtaining original polyurethanes. This approach is safer than conventional polyurethane synthesis, which uses toxic isocyanates.

### 1.3. Polymers Properties Derived from Isosorbide

**Figure 6:**
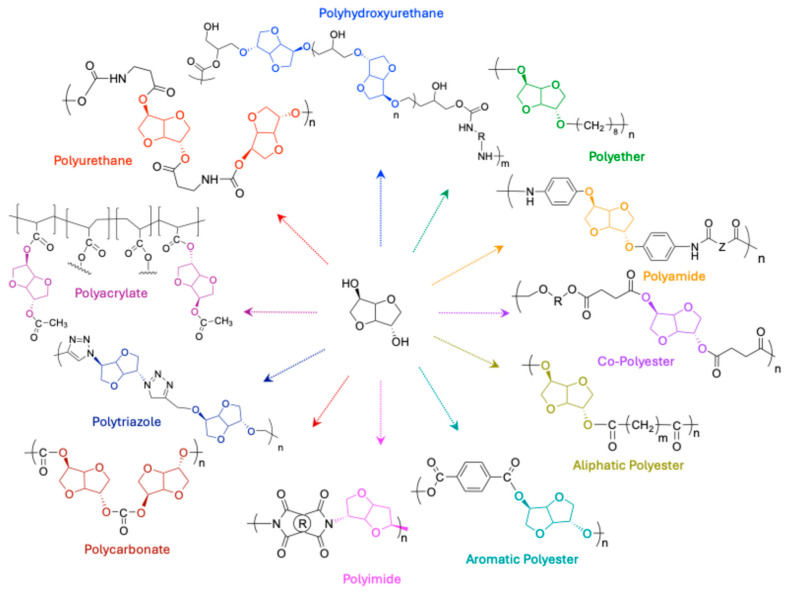
Representation of some polymers derived from isosorbide.

Isosorbide can be used directly or via its derivatives to develop various types of aromatic and aliphatic materials, including polyesters, polyethers, polyurethanes [39,40,41,42,43], and epoxy resins [36,44,45,46], as illustrated in Figure 6.

Numerous studies have emphasized that incorporating isosorbide into a macromolecular chain frequently increases the original polymer’s glass transition temperature (Tg) and thermal stability [47,48,49,50]. The rigid structure and spatial configuration of this compound enabled the development of thermoplastic or thermosetting polymers with properties like those of polymers derived from aromatic structures [4].

Several isosorbide-based polyethers have been described in the literature using the Williamson reaction, by reacting isosorbide or one of its derivatives with a halogenated monomer. The molar masses obtained are significantly low, not exceeding 1000 g/mol [51].

Furthermore, other researchers have successfully demonstrated the synthesis of high molar mass polyacetal using innovative methods such as metathesis [52] or extreme mechanical stirring at low temperatures [53].

Polyamides with atypical optical properties could be produced through the polycondensation of diphenylaminoisorbide (compound 9) with various diacyl chlorides (either aromatic or aliphatic) [24]. They presented high Tg values due to the presence of multiple cyclic structures, including aromatic rings and isosorbide. Notably, with the Z group as hydroquinone, the Tg can soar beyond 300 °C.

Various studies in the literature have examined the development of aromatic and aliphatic polyesters and copolyesters [3,6,54,55]. During these polycondensations, isosorbide was added with diacids at high temperatures with the initial objective of increasing molar masses. However, under these conditions, the obtained molar masses remained low (i.e., between 1000 and 4000 g/mol), probably due to the different reactivity of the hydroxyl groups. However, the use of isosorbide induced an increase in the glass transition temperature of the derived polymers.

Various polyimides were synthesized by condensing isosorbide diamine with dianhydrides derived from petrochemicals [27]. These polyimides have Tg values ranging from 288 °C to 322 °C and exhibit excellent optical transparency. It should be noted that their thermal and mechanical properties are comparable to those of petroleum-derived polyimides.

Linear and cyclic polycarbonates were prepared by reacting isosorbide and its derivative, isosorbide bischloroformate (BIC compound 12). The authors report that low temperatures favor the formation of linear polymers, while high temperatures favor cyclization. On the other hand, utilizing the chemical process of reacting isosorbide with diphosgene and pyridine yields linear polycarbonates with a molar mass of about 50,000 g/mol and a Tg of around 165 °C.

Polytriazoles derived from isosorbide have been developed using diazide and dialkyne precursors (compounds 13 & 14, Figure 5) [26]. All resulting polytriazoles are amorphous with Tg values ranging between 125 °C and 160 °C, while their thermal degradation temperatures span from 325 °C to 347 °C.

Free radical polymerization of acetylated acrylic isosorbide (compound 18, Figure 5) afforded the production of poly(acetylated acrylic isosorbide) (PAAI). This compound underwent controlled radical polymerization (RAFT) with either poly(n-butyl acrylate) (PnBA) or poly(2-ethylhexyl acrylate) (PEHA) to create triblock acrylic copolymers. These copolymers were developed for use as pressure-sensitive adhesives (PSA) [28]. The adhesive characteristics of PAAI-PnBA-PAAI triblocks copolymers were highly satisfactory compared to commercial grades derived from PMMA.

### 1.4. Motivations for Our Research

The paragraphs above demonstrate that isosorbide has been utilized in various applications, ranging from pharmaceuticals to polymers. Its non-toxicity, the differential reactivity of its hydroxyl groups, and the molecular rigidity of its chemical structure have been evidenced in numerous studies. However, some limitations have also been reported regarding the use of isosorbide. For instance, several researchers have shown that achieving high molar mass polymers with a high percentage of isosorbide incorporation is a challenging task due to chain scission and thermal degradation [55,56,57]. In the same studies, the coloring during polymerization is also regarded as an issue. To address this problem, Charbonneau et al. demonstrated that the yellowing of isosorbide-derived PEIT can be reduced by the addition of thermally stable compounds, infrared absorbers, UV stabilizers, dyes, and pigments [58,59].

Table 1 lists a non-exhaustive compilation of studies published by the scientific community that highlight the importance of experimental conditions in reactions involving isosorbide. Many researchers observed the yellowing of isosorbide-derived polymers. However, this phenomenon does not appear to be attributed to the presence of aromatic groups, as two similar structures present quite different aspects (compare entries 1 & 2). The exclusive presence of aliphatic groups is insufficient to prevent the undesired coloring (entry 3). The use of bulk polymerization does not appear to be the solution to this problem (entries 1–6). Very different situations are also described with the syntheses carried out in solvent (entries 7–8). In other words, it seems incorrect to think that the yellowing of certain isosorbide-based polymers and their low molecular weight are two inevitable consequences of using this biobased diol. The only essential parameter that emerges is the combination of time and temperature. Indeed, all experiments carried out at high temperatures and over several hours yield yellow polymers with low molar mass (entries 2–4). A significant reduction in reaction time appears to be an effective way to minimize the risk of yellowing. Still, at the same time, it does not guarantee the formation of large macromolecular chains (entry 1). Furthermore, the use of lower temperatures does not guarantee the absence of coloration when the synthesis time is close to 20 h (entries 6 & 7). Ultimately, the best synthesis conditions seem to be achieved with a moderate synthesis temperature and a limited associated duration.

All of the above surveys suggest that isosorbide exhibits a limited thermostability. On this specific point, the vast majority of authors rely on Fleche’s work [1], which provides the primary characteristics of isosorbide. In this latter research, the melting temperature of isosorbide is evaluated to be between 61 and 64 °C, while its thermal degradation occurs above 270 °C. However, the techniques and experimental conditions used for the corresponding measurements are not described. Surprisingly, no in-depth studies on isosorbide exist in the literature, despite its widespread use in numerous syntheses to produce various (macro)molecules.

It is worth noting that post-synthesis degradation of isosorbide-based polycarbonates has also been reported by Xu et al., who showed that isosorbide-containing copolycarbonates undergo more severe chain scission and yellowing than BPA-based analogs [62]. Nevertheless, the addition of antioxidants can mitigate such effects. This situation differs from the above studies, where the observed yellowing and reduced molar mass occur already during the polymerization and are mainly governed by the combination of time and temperature applied to the monomer.

Although other factors, such as oxidation, comonomer structure, catalyst loading, or isosorbide content, may further influence the extent of yellowing, the comparative overview summarized in Table 1 clearly indicates that reaction time and temperature remain the primary drivers governing discoloration and molar mass reduction across very different isosorbide-based polymers. In fact, the influence of isosorbide content itself reinforces our main conclusion: the isosorbide unit seems to represent a weak link when exposed to excessive time and/or temperature.

On this basis, we conducted a study of the physical and chemical behavior of isosorbide using various complementary methods, including differential scanning calorimetry (DSC), thermogravimetric analysis (TGA), and rheology. Our work also includes the analytical chemistry analysis of this compound. The results are carefully examined and compared to provide a factual discussion and a convincing demonstration based on reliable structure–property relationships.

## 2. Results and Discussion

### 2.1. Chemical Analyses

**Figure 7:**
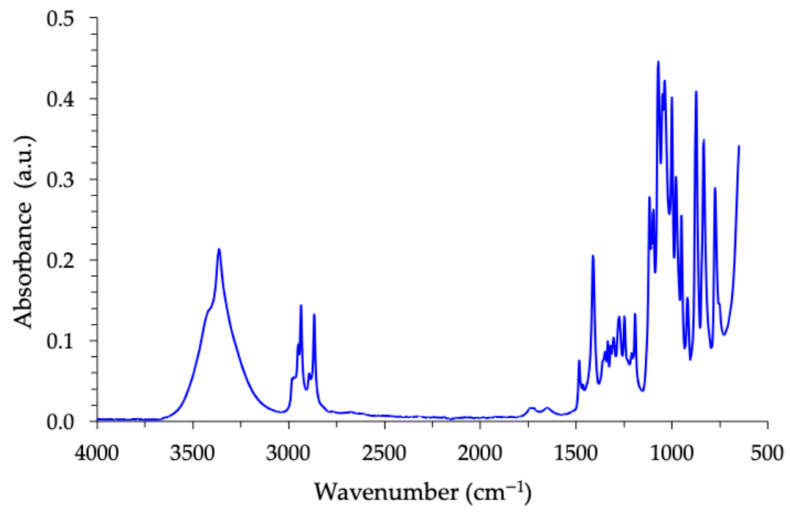
FTIR spectrum of isosorbide measured in absorbance mode.

Before any chemical analysis, isosorbide was stored in a desiccator to prevent any water absorption. The chemical structure of isosorbide was first studied using FTIR spectroscopy. Despite its seemingly simple chemical structure, the FTIR spectrum of isosorbide exhibits multiple absorbance bands (Figure 7). Their interpretation is proposed in Table 2 using different references [63,64,65].

The absorbance bands described above include those at 3430 cm^−1^ and 3370 cm^−1^, which correspond to OH groups in the exo position and endo position, respectively. This differentiation is crucial, as it can be utilized to regulate monofunctionalization and/or polymerization reactions. However, this spectrum is insufficient to state that the compound studied is pure. Then, complementary analyses using ^1^H NMR spectroscopy were performed.

**Figure 8:**
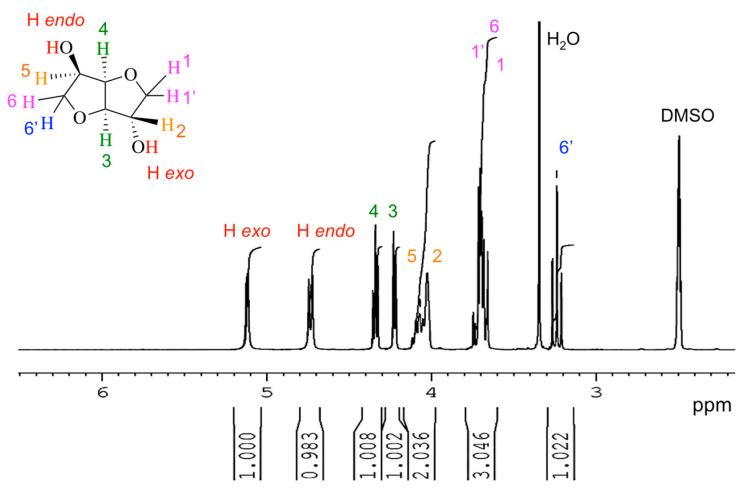
^1^H NMR spectrum of isosorbide (recorded with DMSO-d6 at a frequency of 400 MHz).

The ^1^H NMR spectrum of IS (Figure 8) appears relatively complex. It is similar to that reported in the literature [5,22,66,67,68,69,70,71,72,73]. According to these works, this complexity is inherent in the non-symmetry of the IS molecule. In other words, the stereochemistry, and hence the non-equivalence of various hydrogen atoms, induce this complexity.

Two distinct peaks are associated with the protons of the hydroxyl groups. The proton of the endo group appears at around 4.75 ppm, while that of the exo group is centered at 5.15 ppm [53,74]. This difference is the direct consequence of the two protons being magnetically and electronically non-equivalent. Additionally, the endo proton is involved in an intramolecular hydrogen bond.

After verifying the purity of the isosorbide, the next step was to characterize its physicochemical properties using various techniques.

### 2.2. Differential Scanning Calorimetry (DSC) and X-Ray Diffraction (XRD)

**Figure 9:**
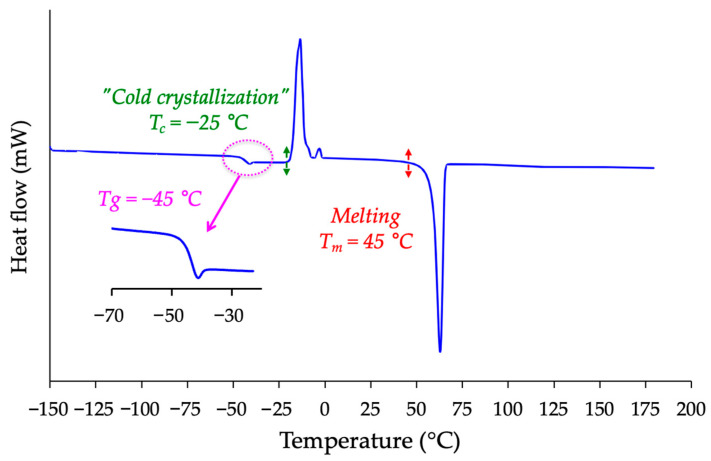
DSC thermogram of isosorbide performed in N_2_ from −150 °C to 200 °C (5 °C/min).

The calorimetric behavior of isosorbide was measured using a differential scanning calorimeter (DSC) from −150 °C to 200 °C under a nitrogen atmosphere with a heating rate of 5 °C/min. All other experimental details are provided in the Materials and Methods section. The resulting thermogram is shown in Figure 9. This thermogram reveals several interesting features. First, an endothermic peak is observed between 45 °C and 62 °C, corresponding to the melting of isosorbide. The onset of melting was consistently detected at approximately 45 °C, i.e., about 15 °C lower than the commonly reported value [1]. This result constitutes clear and reproducible experimental evidence of the melting behavior of isosorbide. In contrast, the values usually cited in the literature (61–64 °C) lack methodological details and are therefore not conclusive. Our determination is based on the onset of fusion, a rigorous thermodynamic criterion, and was performed using a DSC instrument calibrated weekly with indium.

Unexpectedly, the DSC signal also shows a discontinuity at −45 °C, consistent with a glass transition phenomenon. While such behavior is typical of polymers, its occurrence in a small molecule like isosorbide is remarkable. To our knowledge, this singularity has not been previously reported. The associated endothermic overshoot corresponds to enthalpy relaxation, confirming that part of the material can adopt a molecular glass state. Between this transition and the melting peak, an exothermic event was recorded, likely due to the “cold crystallization” of isosorbide.

**Figure 10:**
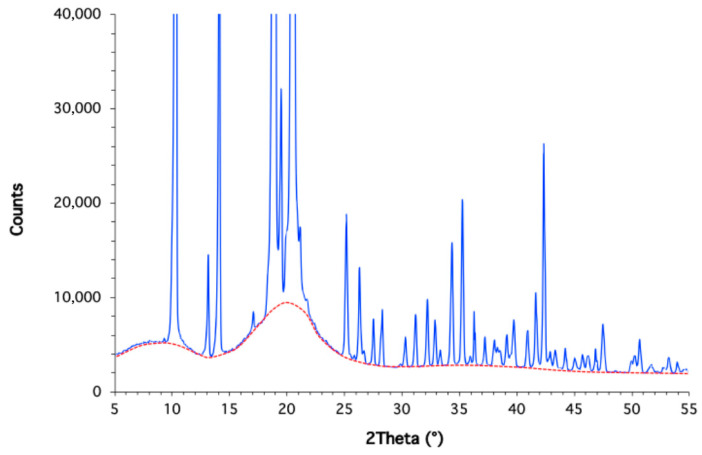
Wide-angle X-ray diffraction (XRD) pattern of isosorbide. The diffractogram shows sharp Bragg reflections from crystalline domains (blue) and a broad diffuse halo (red) from amorphous regions, corresponding to ca. 70% crystallinity and ca. 30% amorphous content.

Altogether, these features strongly resemble the calorimetric behavior of semi-crystalline polymers such as polyethylene terephthalate [75], poly(lactic acid) [76], or poly(aryl ether ether ketone) [77], which exhibit a combination of Tg, cold crystallization, and melting. This analogy refers only to the coexistence of amorphous and crystalline domains and should not be interpreted as evidence that isosorbide is semi-crystalline. To verify this analogy, complementary wide-angle X-ray diffraction (XRD) analyses were performed (Figure 10). The diffractogram exhibits sharp Bragg reflections, characteristic of crystalline domains, together with a broad diffuse halo centered at 20° 2θ, which is the signature of the amorphous regions. Integration of this halo relative to the crystalline reflections yields an estimated crystallinity of about 70%, i.e., approximately 30% of the material exists in an amorphous state. These results are fully consistent with the DSC data. Together, calorimetry and XRD demonstrate that isosorbide cannot be regarded as a purely crystalline molecular solid but instead contains a significant amorphous contribution coexisting with its crystalline lattice, with part of the material behaving as a molecular glass and showing calorimetric signatures analogous to semi-crystalline polymers. This analogy refers only to the coexistence of amorphous and crystalline domains and should not be interpreted as evidence that isosorbide is semi-crystalline.

In the following section, thermogravimetric analyses (TGA) were carried out to characterize the thermal degradation behavior of isosorbide under various conditions.

### 2.3. Thermogravimetric Analysis

The thermostability of isosorbide was evaluated using a thermogravimetric analyzer (TGA). The initial analysis was conducted under an air atmosphere using a thermal ramp with a rate of *β* = 10 °C/min, a rate commonly used in scientific research. Under these experimental conditions, the onset of thermal degradation of isosorbide occurs at 150 °C, corresponding to a mass loss of 5%.

**Figure 11:**
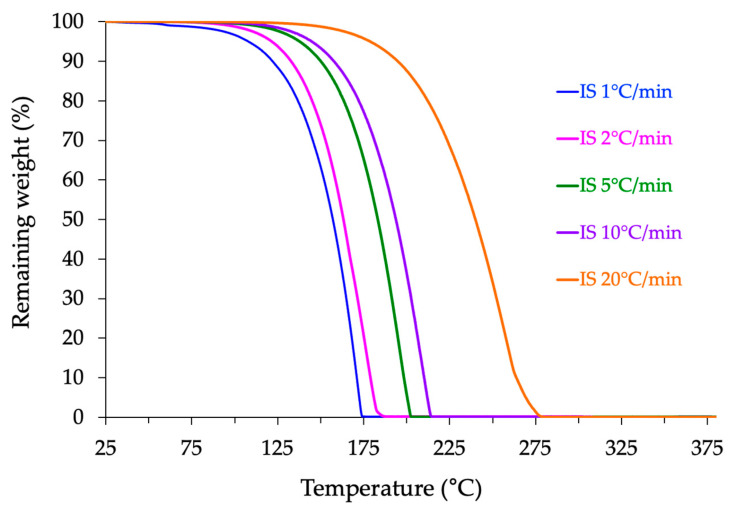
Effect of the thermal ramp on the thermogravimetric profile of isosorbide recorded in air from ambient to 375 °C.

However, when the same experiment is conducted with a new sample and a different temperature ramp, the value of the onset of thermal degradation changes, as depicted in Figure 11.

Then, this critical temperature should be named as the “apparent” degradation temperature, *TdegA*. More specifically, the *TdegA* value decreases as the thermal ramp value *β* decreases. This change can be explained by the fact that an experiment conducted with a lower thermal ramp requires a longer thermal residence time for the sample in the heated measuring chamber of the TGA device. In other words, the kinetics of the degradation process are better detected with a low thermal ramp, while a higher ramp mainly reflects the influence of the thermal factor. Such a multi-rate methodology has been recommended by the ICTAC Kinetics Committee [78] and validated in recent studies on fluoropolymers, which demonstrated that single-rate experiments, especially at 10 or 20 °C/min, can artificially shift degradation onsets to higher values [79,80].

**Figure 12:**
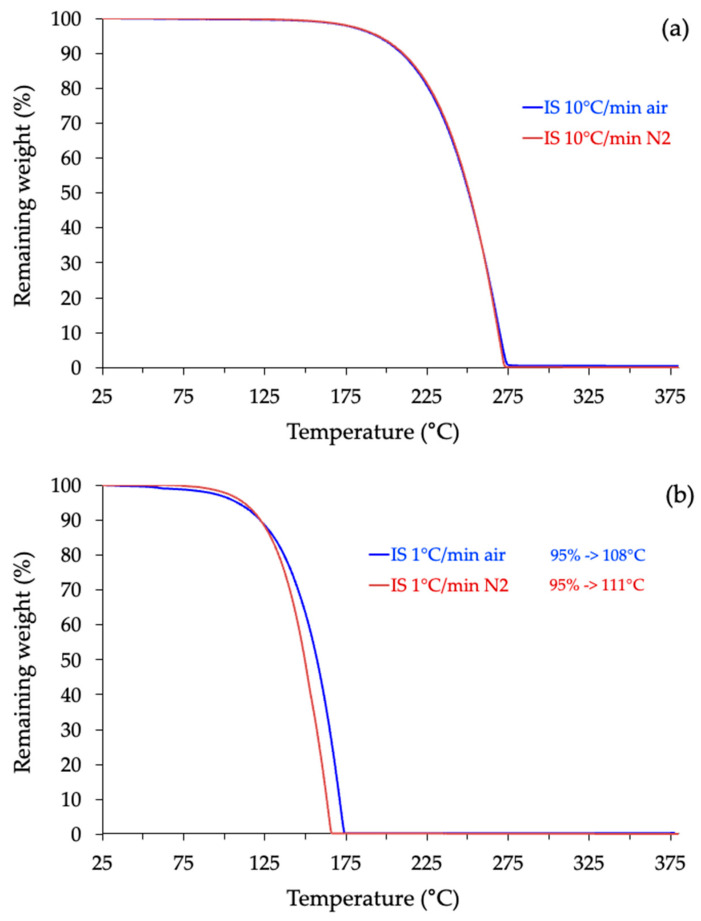
Influence of the atmosphere on the thermogravimetric profile of IS performed under air (blue signal) or inert atmosphere (red) at 10 °C/min (**a**) or 1 °C/min (**b**).

In addition, a comparison between experiments performed under air and under nitrogen confirmed that the atmosphere has only a minor effect at higher heating ramps (≥10 °C/min), where the degradation curves nearly overlap (Figure 12a). Even at the lowest heating rate (1 °C/min), the difference between both atmospheres remains small. However, oxidation effects become slightly more pronounced due to the longer residence time of the sample in the furnace (Figure 12b). This observation confirms the robustness of the air-based measurements while also reflecting realistic processing conditions.

**Figure 13:**
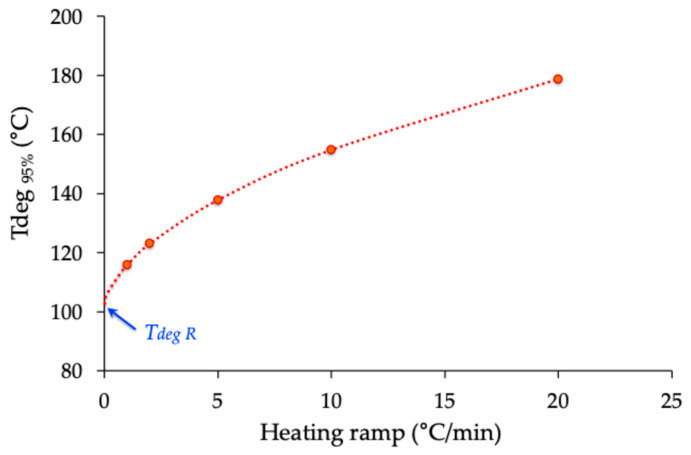
Evolution of the apparent degradation temperature (*T_degA_*) with the thermal ramps used in the thermogravimetric analysis of isosorbide. The dotted line serves as a trend line.

At the extreme limit, a TGA should be conducted at a low heating rate, such as 0.1 °C/min, to account for both the influence of time and temperature on the degradation process. However, such an experiment would take more than two days to record the change in sample mass over the temperature range between 25 and 400 °C. Nevertheless, it is possible to evaluate the accurate degradation temperature using a methodology already developed [81]. It consists of extrapolating the *TdegA* = f(*β*) curve at *β* = 0 (Figure 13). Under these conditions, the real degradation temperature (*TdegR*) of isosorbide is approximately 100 °C, which is 170 °C lower than the value proposed in the literature without any experimental evidence [1,6,25,39,51,53,55,56,57,60,61].

This latter result appears to correspond to the observations previously documented in Section 1.4 regarding the potential origin of the yellowish coloration and reduced mass of various types of isosorbide-derived polymers as described in the literature. It has been hypothesized that the synthesis conditions, in particular the high temperature and duration, were likely responsible for the defects observed in the materials produced under these conditions. The thermal degradation value of isosorbide determined in this study also helps to explain the decrease in thermostability of polyesters prepared with increasing fractions of isosorbide as reported in a recent paper [60].

The TGA investigations established the intrinsic thermal degradation profile of isosorbide across a range of experimental conditions. To complement these results, attention was next directed toward its viscoelastic behavior. Thermomechanical analysis of IS was therefore undertaken to provide additional insight into the molecular mobility and structural organization of this diol, as discussed in the following section.

### 2.4. Thermomechanical Properties

**Figure 14:**
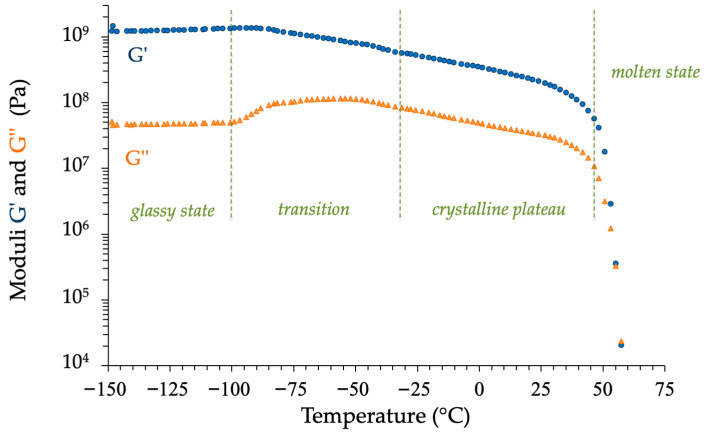
Thermomechanical behavior of isosorbide from −150 °C to 70 °C.

To our knowledge, there are no reports in the literature on the thermomechanical behavior of isosorbide. The evolution of the shear complex modulus of this diol as a function of temperature is presented in Figure 14. The analysis indicates the presence of four distinct thermal domains. The first zone represents the glassy state, where the storage modulus *G*′ (>1 GPa) is greater than the loss modulus *G*″. The second zone shows an increase in the loss modulus *G*″ and a decrease in the storage modulus *G*′ over a temperature range from −100 to −40 °C. This region resembles the phenomenon of mechanical relaxation associated with the glass transition of a polymer [82,83,84]. If this result is somewhat surprising, given that IS is a small molecule that arranges itself in a crystalline phase in the solid state, it agrees with the DSC data already described in this paper. Additionally, between −40 and +45 °C, we observed a gradual but moderate decrease in both components of the complex shear modulus G*, like the crystalline plateau in semi-crystalline polymers. The sharp drop in the two curves indicates the melting of the IS at 45 °C, which is once more consistent with the DSC data. Beyond that point, the loss modulus becomes the predominant component of the complex shear modulus.

**Figure 15:**
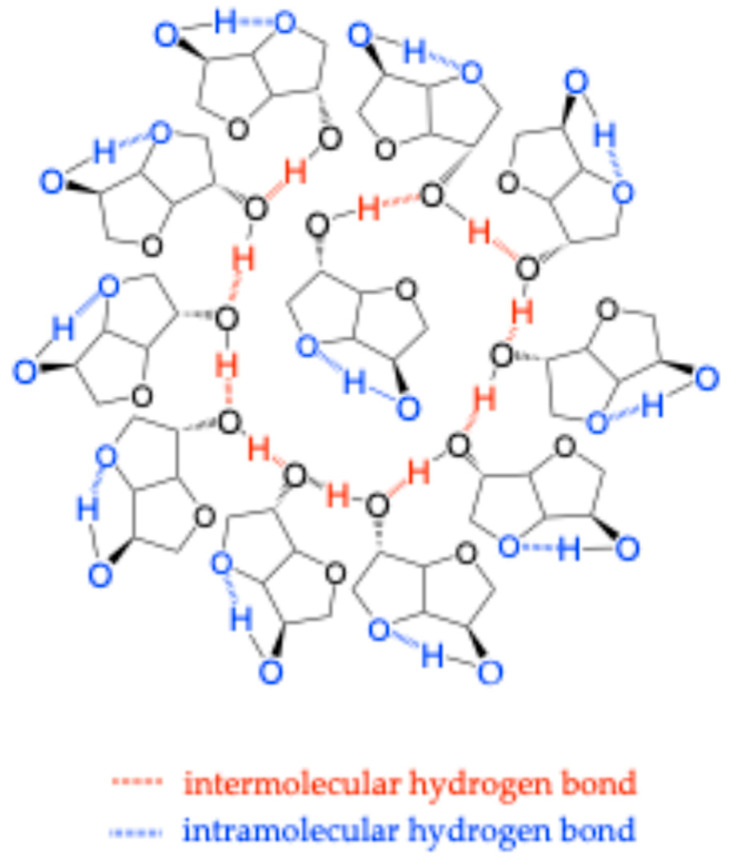
Proposed representation of cooperative motions between isosorbide molecules through their hydrogen bonding.

Our results suggest the presence of a glass transition in IS, as supported by rheometry and DSC data. This implies that the isosorbide molecules are not simply arranged in a crystalline structure. The different endo–exo configurations of the hydroxyl groups are most likely responsible for the intra- and intermolecular interactions in isosorbide. This could explain the presence of cooperative movements involving several molecular units, as sketched in Figure 15. In this approach, the presence of two types of hydrogen bonds likely allows the formation of a pseudo-network, leading to a molecular glass [85,86,87,88]. The intramolecular bond within the same IS molecule locks the isosorbide molecule in a specific conformation, while intermolecular bonds between different IS molecules increase cooperativity. As a result, isosorbide molecules organize themselves into a molecular architecture that can evolve depending on temperature to describe more stable thermodynamic states.

In the representation proposed in Figure 15, the hydrogen bonds are drawn in a unidirectional manner. In other words, they are aligned with the axis of the associated chemical bond. The combined effect of these bonds creates a potential well from which the molecules can be released with a simple increase in temperature, in accordance with Boltzmann’s law. This results in an “apparent glass transition” defined by a critical temperature. Above this transition, the molecules can rearrange themselves to reduce their entropy by forming a crystal.

It should be recalled that all the experimental results reported here (DSC, TGA, XRD, FTIR) were obtained on isosorbide recrystallized by sudden cooling from acetone, as described in Section 3.1. This state ensures purification and reproducibility of the measurements. Although a slower recrystallization process could potentially alter the crystalline fraction, the present study focused on this rapidly recrystallized form, which proved to be the most reliable for comparative analyses. In addition, XRD confirmed that recrystallized isosorbide contains approximately 70% crystalline fraction and approximately 30% amorphous fraction, fully consistent with the calorimetric singularities discussed above. By contrast, the specimens prepared for rheological testing required remelting and subsequent slow cooling to obtain rectangular plates adapted to torsional analysis. This slower cooling likely increased their crystalline fraction compared to the rapidly recrystallized samples, so their thermal history is different and direct comparison with the other measurements must be considered with caution. Altogether, these complementary results provide a coherent picture of isosorbide’s dual nature and its intrinsic limitations, setting the stage for the concluding remarks on its implications for polymer synthesis.

## 3. Materials and Methods

### 3.1. Isosorbide

Isosorbide (CAS 652-67-5) with a purity of 98% was purchased from Merck-Sigma-Aldrich (Darmstadt, Germany). Before use, it was dissolved in acetone at ambient temperature. The recrystallization step was achieved by suddenly exposing the round-bottom flask containing the previous solution to an ice bath. Then, after filtering and drying, this operation allowed the production of a water-free isosorbide powder.

### 3.2. Fourier Transform Infrared (FTIR) Experiment

The chemical structure of isosorbide was first investigated using a Fourier Transform Infrared (FTIR) Spectrum One spectrometer from Perkin Elmer (Waltham, MA, USA). This apparatus was equipped with a Universal Attenuated Total Reflectance (ATR) accessory and a deuterated triglycine sulfate (DTGS) detector. It enabled the recording of the isosorbide chemical spectrum in absorbance mode and by direct deposit on the instrument Zn-Se crystal surface. In other words, no dilution or physicochemical treatment was necessary before analysis. The experiment was performed in the range 4000–650 cm^−1^ with a resolution of 2 cm^−1^ by accumulating 32 scans. The spectra were interpreted using several reference spectral libraries [63,64,65].

### 3.3. Nuclear Magnetic Resonance (NMR) Analyses

Nuclear magnetic resonance (NMR) analysis of isosorbide was performed to complete the chemical characterization of this compound. The isosorbide NMR spectrum was recorded on a Bruker Avance spectrometer (Billerica, MA, USA) operating at 400 MHz (^1^H) and at room temperature in deuterated dimethyl sulfoxide (DMSO-d_6_) as the solvent. The chemical shifts were in parts per million (ppm) and referenced to the peak of residual DMSO-d_6_ at *δ* = 2.50 ppm.

### 3.4. Differential Scanning Calorimetry (DSC) Analyses

The thermal properties of isosorbide were investigated using a StarOne Differential Scanning Calorimeter (DSC, from Mettler Toledo, Greifensee, Switzerland) in dynamic mode under a nitrogen flow rate of 50 mL min^−1^. The DSC experiments were carried out over the temperature range −50 °C to 250 °C, with a heating rate of 5 °C/min. The instrument was calibrated weekly using high-purity indium standards, ensuring accurate temperature and enthalpy measurements. Each experiment was repeated on independent samples, and the observed transitions (glass transition, cold crystallization and melting) were consistently reproduced, confirming the robustness of the results.

### 3.5. Thermogravimetric Analyses (TGA)

The thermostability of isosorbide was evaluated using a TGA2 thermogravimetric analyzer from Mettler Toledo (Greifensee, Switzerland) in dynamic mode, under both air and nitrogen atmospheres (flow rate: 50 mL·min^−1^). Analyses were conducted from 25 °C to 400 °C at multiple heating rates (β = 1, 2, 5, 10, and 20 °C·min^−1^). The instrument was routinely calibrated for temperature accuracy using Curie-point standards, ensuring accurate onset determination. Each measurement was repeated on independent samples, and the degradation profiles obtained under identical conditions were found to be highly reproducible. The adoption of multiple heating rates follows the recommendations of the ICTAC Kinetics Committee [78] and prevents the artificial overestimation of thermal stability often observed in single-rate TGA experiments [79,80].

### 3.6. Thermomechanical Tests

**Figure 16:**
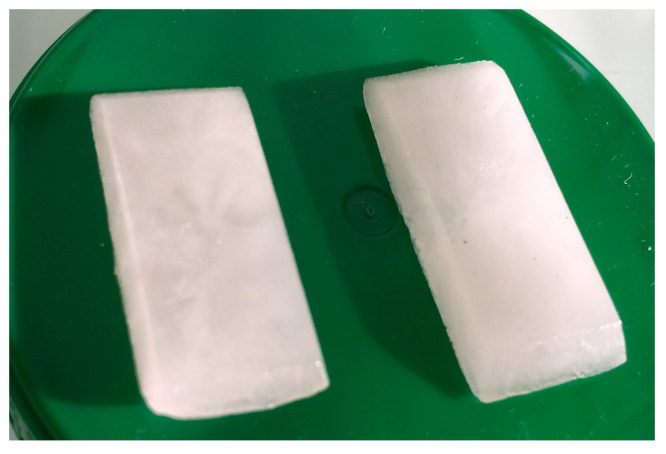
Picture of molded isosorbide samples for further thermomechanical tests (38 × 12 × 3 mm^3^).

Isosorbide, initially in the form of a fine powder, was molded into parallelepiped samples (35 mm × 10 mm × 2 mm) using a heated hydraulic press set at the temperature *T* = 60 °C. The obtained platelets, illustrated in Figure 16, could be analyzed using dynamic rheometry with a stress-controlled rheometer AR2000Ex from TA Instruments (New Castle, TX, USA) equipped with a rectangular torsion geometry.

The rheological tests were performed in air at a heating rate of 3 °C/min within a temperature range of −150 °C to 70 °C. The applied strain was set at 0.1% after determining the linear rheology range. The angular shear frequency was kept constant (*ω* = 1 rad/s).

### 3.7. X-Ray Diffraction Experiments

X-ray diffraction (XRD) analyses were performed at room temperature on a Philips X’Pert PRO MPD diffractometer (Philips, Amsterdam, The Netherlands) in Bragg–Brentano geometry, using Cu Kα radiation (λ = 1.5406 Å). The instrument was equipped with a θ–θ goniometer, a spinner-type sample holder and an X’Celerator linear detector. Isosorbide samples were prepared on zero-background sample holders to minimize parasitic scattering. Diffraction patterns were collected in the 2θ range of 5–55° with a step size of 0.033° 2θ, over a total acquisition time of 1 h. The diffractograms were subsequently analyzed to separate crystalline reflections from the amorphous halo, allowing for a semi-quantitative estimation of the amorphous fraction.

## 4. Conclusions

This study conducted an in-depth investigation of the thermal and rheological properties of isosorbide, a biobased diol that has been of interest to the scientific and industrial communities for over 50 years. Unlike previous research, which often used isosorbide without prior in-depth characterization, this study reveals fundamental aspects of this compound. In particular, it shows that the real degradation temperature of isosorbide is significantly lower than previously reported in the literature (about 100 °C instead of 270 °C). This value was established through multi-rate TGA experiments performed under both air and nitrogen atmospheres, in accordance with ICTAC recommendations, which ensured a robust determination of intrinsic stability.

Our research also demonstrates that isosorbide exhibits remarkable calorimetric and viscoelastic behaviors, with features typically associated with systems that combine crystalline and amorphous domains. DSC revealed a glass transition at −45 °C, followed by cold crystallization and melting at approximately 45 °C. X-ray diffraction confirmed this duality by showing sharp Bragg peaks superimposed on an amorphous halo, allowing a degree of crystallinity of around 70% to be estimated. To our knowledge, such properties have not been reported before in the literature. They can be attributed to intra- and intermolecular interactions facilitated by hydrogen bonds, supporting the notion that part of isosorbide behaves as a molecular glass.

The importance of this research lies in its contribution to a more complete and accurate understanding of the fundamental properties of isosorbide, which is crucial for the development of new materials and functional molecules. Considering the data obtained in this work, one potential perspective of this study is to synthesize polymers already described in the literature or, conversely, new macromolecules, but under milder conditions. In all cases, a significant portion of the research effort should be devoted to optimizing polymerization protocols to minimize thermal degradation and preserve the properties of the final materials.

## Figures and Tables

**Table 1:** Research showing how synthesis conditions affect the molar mass and color of some polymers based on isosorbide.

N°	Polymer	Synthesis Conditions	T (°C)	Time (h)	Mn (g/mol)	Color	Refs.
1	Poly(isosorbide-terephthalate)	in bulk	180	0.17	3000	colorless	[56]
2	Poly(ethylene-co-isosorbide) terephthalate	in bulk	180–265	4	4000	yellow	[60,61]
3	Poly(isosorbide-succinate)	in bulk	180	4	2000	yellow	[55,57]
4	Polycarbonate	in bulk with diphenyl carbonate	160–245	4	3200	yellow	[6]
5	Polycarbonate	in bulk with diphosgene and pyridine	20	24	50,000	colorless	[25]
6	Polyurethane	in bulk	70	24	50,000	colorless	[39]
7	Aromatic and linear polyether	solid–liquid two phases mixing	80	4–20	300–1000	brown	[51]
8	Aliphatic polyacetal	solvent (DMSO)	90	0.08	9000	colorless	[53]

**Table 2:** Position and attribution of isosorbide FTIR absorbance bands.

Wavenumber (cm^−1^)	Assignment	Notation	Intensity
3600–3200	O–H stretching (H bonded)	ν(OH)	br, s
2970–2940	CH stretching (aliphatic, CH/CH_2_)	ν(CH)	m
2945–2905	Asymmetric CH_2_ stretching	ν_as(CH_2_)	m
2870–2840	Symmetric CH_2_ stretching	ν_s(CH_2_)	m
1640–1610	H–O–H bending (reduced traces of moisture)	δ (H–O–H)	w
1430–1460	HCH bending	δ(HCH)	m
1410 ± 20	In place O–H bending	δ(COH)	w–m
1400–1300	CH_2_ wagging/twisting	ρ/τ(CH_2_)	w–m
1200–1140	C–O–C stretching (asymmetric)	ν_as(C–O–C)	s
1080–1040	C–O–C stretching (symmetric)	ν_s(C–O–C)	m–s
1070–1020	C–O stretching (secondary alcohol)	ν(C–O)	m–s
1000–920	Ring/skeletal C–C–O and C–C modes	ν(skeletal)	w–m
900–650	Out of plane C–H bends and rocking	γ(C–H)	s

Vibration mode: ν = stretching; δ = in plane bending; γ = out-of-plane bending; ρ/τ = rocking/twisting; Intensity: s = strong; m = medium; w = weak; br = broad.

## Data Availability

The data will be provided upon request.
